# Mistaken Diagnoses

**Published:** 1901-02-23

**Authors:** 


### MISTAKEN DIAGNOSES.

The difficulties in the way of making an exact diagnosis in regard to some of the eruptive fevers are often very considerable. According to the last report of the Metropolitan Asylums Board, during the course of the year 1899, no fewer than 1,583 patients, or a percentage on the total admission of 6·3, were after admission found not to be suffering from the diseases mentioned in the medical certificates upon which they were removed to hospital. Of the cases sent in as scarlet fever 53 turned out to be measles, 25 German measles, 100 "erythema," and 120 toncillitis, so that much as our patients would like us to say off-hand what is the matter with them, it is clear enough that mistakes are more difficult to avoid than some people imagine. Typical cases are easily diagnosed. It is from the frequency with which abnormal and non-typical cases occur that the trouble arises. How far cases may diverge from the normal is well illustrated by the published accounts of the recent illness of the Duke of Cornwall and York. According to the books the symptoms which precede the outcoming of the rash in German measles are "short and slight," their duration is "not more than twelve hours," and "rarely in any case is the rash delayed beyond the second day;" while in ordinary measles, as is well known, they last from three to four days, and are often severe, the air passages generally being involved, bronchitis being usually marked, broncho-pneumonia being not uncommon, the tongue being furred, the appetite poor, and the temperature considerably raised. This being so, let us compare the symptoms which we are told occurred in the case of the Duke of Cornwall and York's German measles. He began to be ill on January 24th, when he took to his bed, but it was not until January 29th that a slight redness appeared on the forehead. Meanwhile his symptoms were so far from slight that they included a temperature of 102° F., a troublesome and almost continuous cough, a furred tongue, a friction sound at the base of the left lung, bad nights, restlessness and discomfort. Take another symptom: enlargement of the glands occurs in both forms of measles. In the German form this enlargement "is always an early symptom, and may be detected as long as a week before the rash appears" (Allbutt's system), while in ordinary measles the enlargement is not marked early in the course of the disease. In the Duke's German measles it seems that it was not until January 30th, one day after the rash was first noted, that one gland on each side was found to be enlarged. In German measles the rash is not accompanied with any itching, and in any given area "fading is seldom delayed beyond twelve hours," while in ordinary measles, as is well known, the rash goes on spreading for about three days after it first shows itself, and as a rule the height of the disease roughly corresponds with the time of the full development of the rash. In the Duke's case we are told that the rash first appeared on the 29th; that on the 30th the blush on the forehead was more marked, while on the 31st the rash was profuse, elevated, and very sensitive to the touch; and that during the interval between the 29th and 31st the cough continued to be troublesome, the temperature was 102° F., nausea was present, air entered the chest badly, he complained of being unable to draw his breath, and respiration was almost entirely abdominal; he had slight delirium and occasional vomiting. Evidently he was very ill. From the third day of the rash he rapidly improved. It is to be noted that in drawing this comparison we have compared the symptoms reported to have been observed in the case of the Duke of Cornwall and York with those met with in what may be termed typical cases of German and ordinary measles. It will also be noted how very markedly the attack, so far as the general symptoms are concerned, resembled the ordinary rather than the German form. Yet those who saw the whole case, including the rash, held it to be a severe case of German measles. Do not let it be imagined that we question the diagnosis; but we do say that when unfortunate East End medical men send cases into hospital wrongly diagnosed, it is somewhat hard that they should be exposed to ridicule at the hands of hospital officials; that they should be thus discredited in the eyes of their patients and their friends; and that scorn should be thrown upon them in medical reports, when diagnosis is surrounded with so many chances of error. Let us remember the extraordinary difficulties under which many cases are investigated in the homes of the poor; let us recall the many curious vagaries of disease and the wide divergencies from the normal type which individual cases may show (of which the malady from which the Duke of Cornwall and York has lately suffered gives good examples), and let us deal charitably with those who now and again make "mistaken diagnoses."

